# Further Characterization of Intrastriatal Lipopolysaccharide Model of Parkinson’s Disease in C57BL/6 Mice

**DOI:** 10.3390/ijms22147380

**Published:** 2021-07-09

**Authors:** Isaac Deng, Frances Corrigan, Sanjay Garg, Xin-Fu Zhou, Larisa Bobrovskaya

**Affiliations:** 1 Health and Biomedical Innovation, Clinical and Health Sciences, University of South Australia, Adelaide 5000, Australia; denbi002@mymail.unisa.edu.au (I.D.); Sanjay.Garg@unisa.edu.au (S.G.); Xin-Fu.Zhou@unisa.edu.au (X.-F.Z.); 2 Medical Sciences, University of Adelaide, Adelaide 5000, Australia; Frances.Corrigan@adelaide.edu.au

**Keywords:** Parkinson’s disease, intrastriatal, inflammation, olfactory bulb, colon

## Abstract

Parkinson’s disease (PD) is the most common movement disorder, characterized by progressive degeneration of the nigrostriatal pathway, which consists of dopaminergic cell bodies in substantia nigra and their neuronal projections to the striatum. Moreover, PD is associated with an array of non-motor symptoms such as olfactory dysfunction, gastrointestinal dysfunction, impaired regulation of the sleep-wake cycle, anxiety, depression, and cognitive impairment. Inflammation and concomitant oxidative stress are crucial in the pathogenesis of PD. Thus, this study aimed to model PD via intrastriatal injection of the inflammagen lipopolysaccharide (LPS)to investigate if the lesion causes olfactory and motor impairments, inflammation, oxidative stress, and alteration in synaptic proteins in the olfactory bulb, striatum, and colon. Ten µg of LPS was injected unilaterally into the striatum of 27 male C57BL/6 mice, and behavioural assessment was conducted at 4 and 8 weeks post-treatment, followed by tissue collection. Intrastriatal LPS induced motor impairment in C57BL/6 mice at 8 weeks post-treatment evidenced by reduced latency time in the rotarod test. LPS also induced inflammation in the striatum characterized by increased expression of microglial marker Iba-1 and astrocytic marker GFAP, with degeneration of dopaminergic neuronal fibres (reduced tyrosine hydroxylase immunoreactivity), and reduction of synaptic proteins and DJ-1 protein. Additionally, intrastriatal LPS induced inflammation, oxidative stress and alterations in synaptic proteins within the olfactory bulb, although this did not induce a significant impairment in olfactory function. Intrastriatal LPS induced mild inflammatory changes in the distal colon, accompanied by increased protein expression of 3-nitrotyrosine-modified proteins. This model recapitulated the major features of PD such as motor impairment and degeneration of dopaminergic neuronal fibres in the striatum, as well as some pathological changes in the olfactory bulb and colon; thus, this model could be suitable for understanding clinical PD and testing neuroprotective strategies.

## 1. Introduction

Parkinson’s disease (PD) is the most common movement disorder, characterized by core motor symptoms such as resting tremors, rigidity, bradykinesia, and postural instability [1,2]. These manifestations are predominately a result of progressive degeneration in the nigrostriatal pathway, which consists of dopaminergic cell bodies in the substantia nigra (SN), and their neuronal projections to the striatum [1,2]. PD is also associated with non-motor symptoms such as olfactory dysfunction, gastrointestinal dysfunction, impaired regulation of the sleep-wake cycle, anxiety, depression, and cognitive impairment; however, the pathological processes responsible for these complications are not well understood [1,3].

Another major neuropathological hallmark of PD is the presence of Lewy bodies/Lewy neurites in the surviving dopaminergic neurons, consisting of abnormal aggregates of α-synuclein protein [4]. α-Synuclein protein plays a major role in synaptic function, and alterations in this protein lead to synaptic dysfunction [4,5,6,7]. The α-synuclein protein interacts with vesicle-associated membrane protein 2 (VAMP2) to promote the assembly of the SNARE complex. The SNARE complex is formed by VAMP2, syntaxin and synaptosomal associated protein 25 (SNAP-25), which regulates docking and fusion of synaptic vesicles at the presynaptic membrane [8,9]. Moreover, vesicular monoamine transporter 2 (VMAT2) is critical for the assembly of cytosolic monoamines into synaptic vesicles. It has been reported that VMAT2 levels are reduced in the striatum and SN of PD patients [10,11,12,13]. Other proteins contributing to synaptic dysfunction in PD include deglycase DJ-1 (DJ-1), Parkin and PTEN induced putative kinase 1 (PINK1) [5,14]. DJ-1, Parkin and PINK1 are associated with hereditary PD and they are also important for mitochondrial function and protection against oxidative stress [5]. Thus, we aimed to investigate some of these proteins in this study.

Neurotoxin and genetic-based models have been invaluable in understanding the aetiology of PD, and have illustrated the importance of inflammation and oxidative stress in the disease process [15]. Microglia cells are the prominent mediators of neuroinflammation, and indeed, activated microglia cells have been identified in the SN of post-mortem brain samples of PD patients [16]. Moreover, astrogliosis is evident in the SN of PD patients [17,18]. Inflammatory cytokines and free radicals produced by activated microglia and astrocytes under pathological conditions can induce the expression of enzymes involved in the secretion of potent free radicals such as nitric oxide and superoxide [17,18]. The aforementioned free radicals can react together to form peroxynitrite, which is involved in the nitration of free tyrosine residues on proteins and can be assessed by examining the expression of 3-nitrotyrosine (3-NT)-modified proteins [19,20]. Neuroinflammation can also affect the levels of brain-derived neurotrophic factor (BDNF), a protein essential for differentiation, proliferation, survival, and synaptic plasticity in the CNS. Indeed, it has been reported that BDNF mRNA and protein are reduced in the SN and serum of PD patients compared to healthy controls [21,22]. 

Furthermore, it has been reported that PD is linked to gut inflammation evident by increased tumour necrosis factor-α, interferon-γ, interleukin 6, and interleukin 1β, as well as glial markers such as GFAP and Sox-10 in the colon [23]. The majority of the gut microbiota resides in the colon, and the epithelium lining of the intestines acts as a protective barrier separating the commensal microbes in the lumen of the intestines from the immune system and enteric nervous system (ENS) in the underlying tissue [23,24,25]. Interestingly, it has been reported that PD patients have increased intestinal permeability, which can result in the exposure of ENS to pro-inflammatory products from the lumen. In turn, the pro-inflammatory products could drive inflammation and oxidative stress in the gut, which is evident to induce α-synuclein aggregation and indeed, α-synuclein-positive Lewy bodies are evident in the colon of PD patients [24,25,26]. Of note, an increased risk of PD has been reported in patients with inflammatory bowel disease and it is evident that gut inflammation in these patients is mainly mediated by T-lymphocytes [27,28].

The inflammatory aspects of PD can be mimicked in animal models via the administration of lipopolysaccharide (LPS), which is a component of the cell wall of Gram-negative bacteria and a potent activator of microglial cells via Toll-like receptor 4 [29]. In vivo, LPS can be administered stereotaxically, intraperitoneally and intranasally, and these models can recapitulate features of clinical PD such as degeneration of nigrostriatal pathway, motor impairment and α-synuclein pathology [29,30,31,32,33,34,35]. Localized lesions to the nigrostriatal pathway that replicate features of PD can be generated via intranigral and intrastriatal administration of LPS [29,30,31,32]. It has been hypothesized with some evidence that degeneration of the nigrostriatal pathway starts at the axonal terminals (located in the striatum) and progresses retrogradely to the SN, and the intrastriatal LPS model helps to elucidate this hypothesis [29,36]. Previous intrastriatal LPS models have only explored the major neuropathological hallmarks of PD and the resultant motor complications; however, there is limited evidence on the abnormalities in synaptic proteins, and proteins linked to genetic PD (e.g., Parkin, DJ-1) in the striatum. Moreover, pathological changes in other regions such as the olfactory bulb and colon have not been well examined in intrastriatal LPS models, and these are crucial aspects of PD. Therefore, this study aimed to further characterize the intrastriatal LPS model of PD including motor and non-motor symptoms and associated pathology in the striatum, olfactory bulb, and colon.

## 2. Results

### 2.1. Effects of Intrastriatal Injection of LPS on Motor Behaviour, Olfactory Function and Mood in C57BL/6 Mice

**Figure 1:**
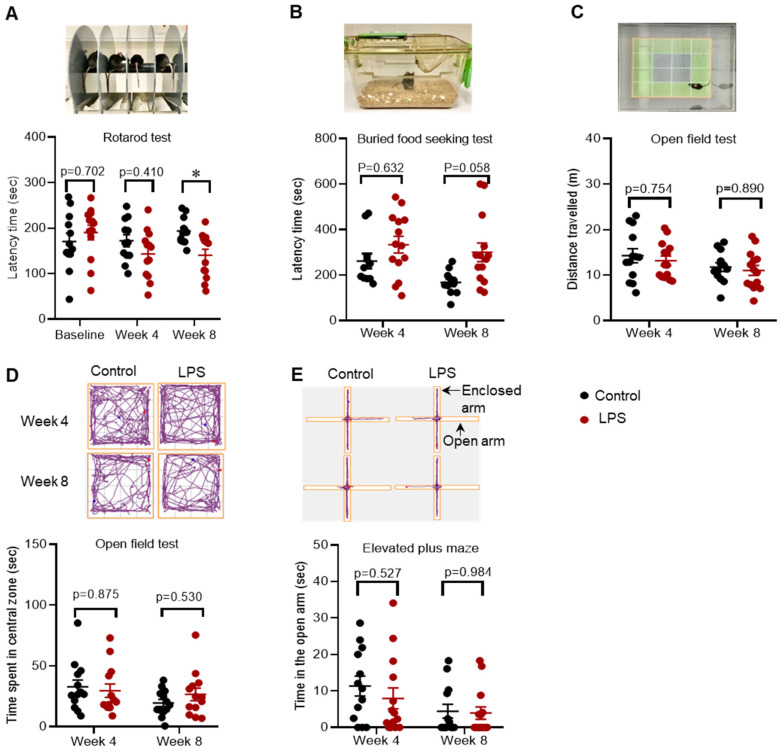
Administration of LPS into the striatum induced motor impairment, but not olfactory dysfunction or anxiety-like behaviour in C57BL/6 mice. (**A**) Set up and latency time in the rotarod test. (**B**) Set up and latency time in the buried food-seeking test. (**C**) Setup and distance travelled in the open field test. (**D**) Computerized tracing of the mice in the open field and time spent in the central zone of the open field. (**E**) Computerized tracing of the mice in the elevated plus-maze and time spent in the open arm. The results are presented as mean ± SEM (control n = 12–13; LPS n = 13–14). Statistical analysis was performed with a two-way ANOVA test, *p* < 0.05 (*).

The rotarod test, which measures motor behaviour, showed that there was no difference in latency time in the LPS group compared to control at 4 weeks post-treatment; however, the latency time was significantly decreased by 27.4% in the LPS group compared to control at 8 weeks post-treatment, indicative of impaired motor function (baseline: 190.31 versus 170.42 s, *p* = 0.702; week 4: 143.23 versus 172.06 s, *p* = 0.410; week 8: 140.36 versus 193.47 s, *p* = 0.033) (Figure 1A).

Intrastriatal LPS did not induce a significant change in latency time in the buried food-seeking test at 4 and 8 weeks post-treatment compared to the control group, indicating no differences in olfactory function (week 4: 333.57 versus 262 s, *p* = 0.633; week 8: 300.36 versus 167.64 s, *p* = 0.058) (Figure 1B).

Furthermore, there was no difference in the time spent exploring the central zone of the open field in the LPS group compared to the control group at 4 and 8 weeks post-treatment (week 4: 29.58 versus 32.79 s, *p* = 0.875; week 8: 26.6 versus 19.53 s, *p* = 0.531) (Figure 1D), and these findings were consistent with no change in time spent exploring the open arm of elevated plus maze (week 4: 7.96 versus 11.35 s, *p* = 0.527; week 8: 3.94 versus 4.45 s, *p* = 0.985) (Figure 1E). Collectively, the results for the open field and the elevated plus-maze test showed that intrastriatal administration of LPS did not induce anxiety like-behaviour.

### 2.2. Administration of LPS Altered the Expression of TH, α-Synuclein and Synaptic Proteins in the Striatum of C57BL/6 Mice

**Figure 2:**
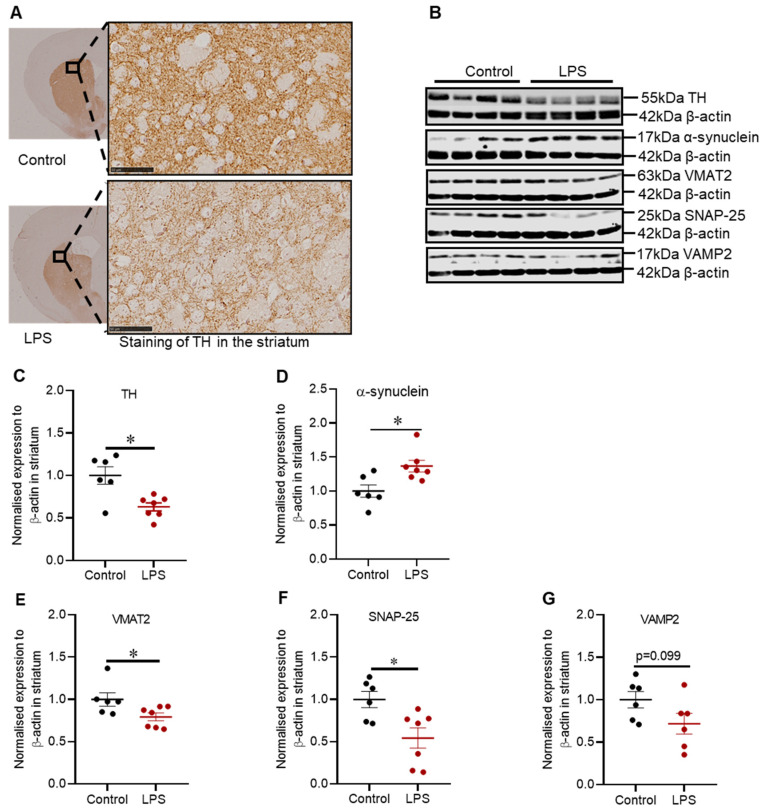
Administration of LPS altered the expression of TH, α-synuclein and synaptic proteins in the striatum of C57BL/6 mice. (**A**) Representative images for immunohistochemical detection of TH in the striatum. (**B**) Representative immunoblots for TH, α-synuclein, VMAT2, SNAP-25 and VAMP2 proteins in the striatum. (**C**) Densitometric analysis of TH protein. (**D**) Densitometric analysis of α-synuclein protein. (**E**) Densitometric analysis of VMAT2 protein. (**F**) Densitometric analysis of SNAP-25 protein. (**G**) Densitometric analysis of VAMP2 protein. The results are presented as mean ± SEM (control n = 6; LPS n = 6–7). Statistical analysis was performed with a Mann–Whitney test, *p* < 0.05 (*). Immunohistochemical detection of TH (scale bar: 50 µm; microscopic magnification: X400) (control n = 5; LPS n = 5).

**Figure A1:**
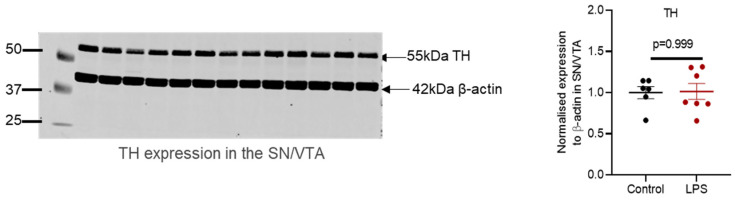
Western blot detection of tyrosine hydroxylase (TH) in the substantia nigra/ventral tegmental area (SN/VTA).

Intrastriatal administration of LPS induced degeneration of dopaminergic neuronal fibres in the striatum depicted by reduced immunoreactivity for tyrosine hydroxylase (TH) (Figure 2A), and this finding was congruent with the reduced expression of TH protein (1.59-fold) in the striatum (*p* = 0.022) (Figure 2C). TH protein was also assessed in the SN (refer to Figure A1 in Appendix A), but there was no significant difference, suggesting that intrastriatal LPS did not induce degeneration in the SN.

Intrastriatal LPS increased the expression of α-synuclein protein by 1.37-fold (*p* = 0.022) (Figure 2D), which was accompanied by a 1.26-fold decreased in VMAT2 protein (*p* = 0.035) (Figure 2E) and a 1.81-fold decreased in SNAP-25 (*p* = 0.022) (Figure 2F) with no significant decrease in VAMP2 protein (*p* = 0.180) (Figure 2G).

### 2.3. Effects of LPS on the Inflammatory and Oxidative Stress Markers, and Proteins Involved in Defence Mechanisms against Oxidative Stress in the Striatum of C57BL/6 Mice

**Figure 3:**
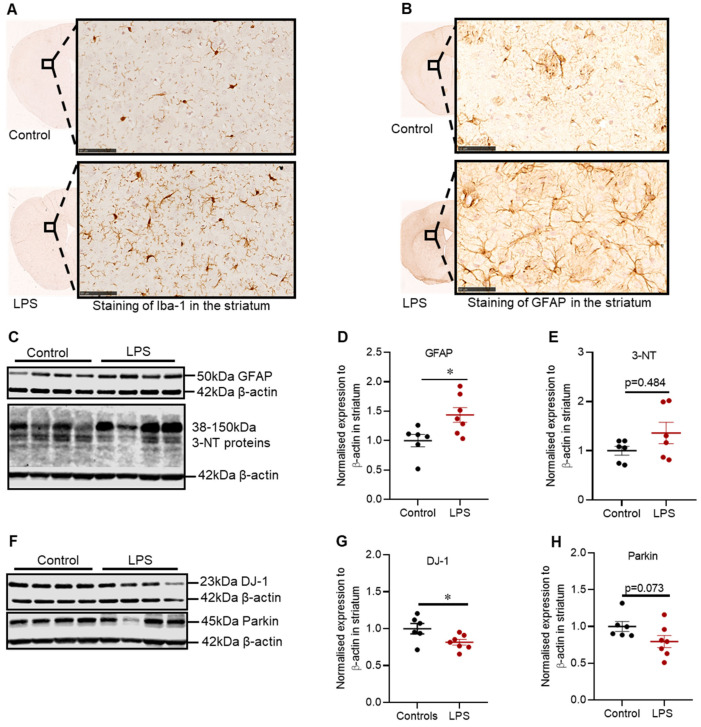
LPS altered the inflammatory and oxidative stress markers, and proteins involved in defence mechanisms against oxidative stress in the striatum of C57BL/6 mice. Representative images for immunohistochemical detection of Iba-1(**A**) and GFAP(**B**) in the striatum. (**C**) Representative immunoblots for GFAP and 3-NT proteins in the striatum. (**D**) Densitometric analysis of GFAP protein. (**E**) Densitometric analysis of 3-NT proteins. (**F**) representative immunoblots for DJ-1 and Parkin in the striatum (involved in defence against oxidative stress). (**G**) Densitometric analysis of DJ-1 protein. (**H**) Densitometric analysis of parkin protein. The results are presented as mean ± SEM (control n = 6; LPS n = 6–7). Statistical analysis was performed with a Mann–Whitney test, *p* < 0.05 (*). Immunohistochemical detection of Iba-1 and GFAP (scale bar: 50 µm; microscopic magnification: X400) (control n = 5; LPS n = 5).

Intrastriatal administration of LPS induced inflammation in the striatum, which was characterized by increased Iba-1 positive microglia cells (Figure 3A) and GFAP positive astrocytes (Figure 3B), and these findings were consistent with the increased expression of GFAP protein in the striatum (1.44-fold; *p* = 0.022) (Figure 3D).

LPS also significantly decreased the protein expression of striatal DJ-1 by 1.23-fold (*p* = 0.036) (Figure 3G), with no significant decrease in the expression of Parkin protein (*p* = 0.073) (Figure 3H); or in 3-NT, a marker of oxidative stress (*p* = 0.484) (Figure 3E).

### 2.4. Intrastriatal Administration of LPS Altered the Expression of TH and Synaptic Proteins in the Olfactory Bulb of C57BL/6 Mice

**Figure 4:**
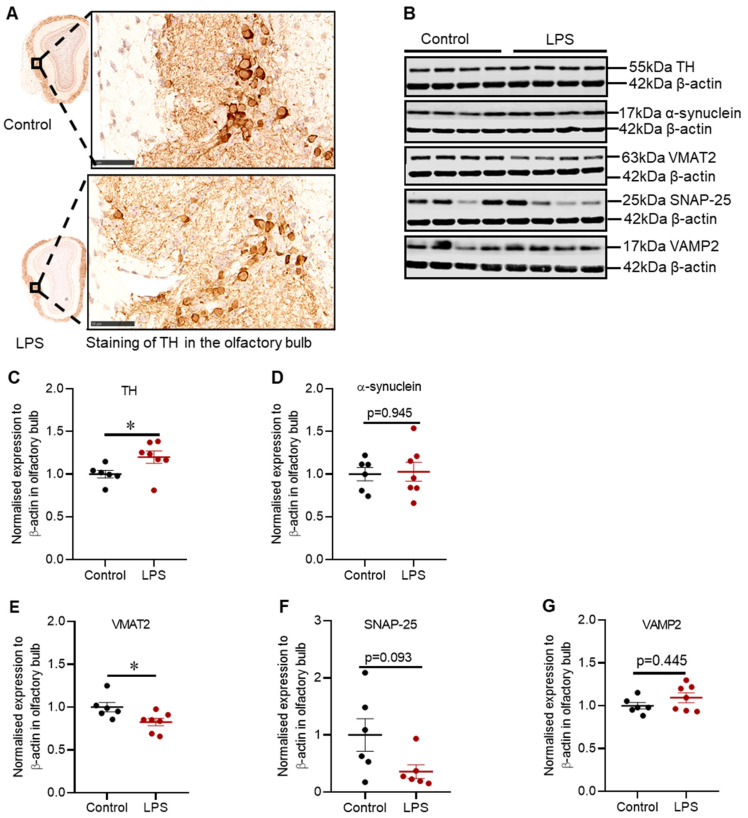
Intrastriatal administration of LPS altered the expression of TH and synaptic proteins in the olfactory bulb of C57BL/6 mice. (**A**) Representative images for immunohistochemical detection of TH in the olfactory bulb. (**B**) Representative immunoblots for TH, α-synuclein, VMAT2, SNAP-25 and VAMP2 proteins in the olfactory bulb. (**C**) Densitometric analysis of TH protein. (**D**) Densitometric analysis of α-synuclein protein. (**E**) Densitometric analysis of VMAT2 protein. (**F**) Densitometric analysis of SNAP-25. (**G**) Densitometric analysis of VAMP2. The results are presented as mean ± SEM (control n = 6; LPS n = 6–7). Statistical analysis was performed with a Mann–Whitney test, *p* < 0.05 (*). Immunohistochemical detection of TH (scale bar: 50 µm; microscopic magnification: X400) (control n = 5; LPS n = 5).

There was no difference in TH positive cells in the glomerular layer of the olfactory bulb between control and LPS treated mice based on immunohistochemical staining (Figure 4A); however, the expression of TH protein was significantly increased by 1.2-fold (*p* = 0.035) (Figure 4C), without alteration in α-synuclein protein (*p* = 0.945) (Figure 4D).

Intrastriatal LPS significantly decreased the expression of VMAT2 protein by 1.2-fold (*p* = 0.0140), with no significant change in SNAP-25 (*p* = 0.093) (Figure 4F) or VAMP2 protein (*p* = 0.445) (Figure 4G).

### 2.5. Effects of LPS on the Inflammatory and Oxidative Stress Markers, and Proteins Involved in Defence Mechanisms against Oxidative Stress in the Olfactory Bulb of C57BL/6 Mice

**Figure 5:**
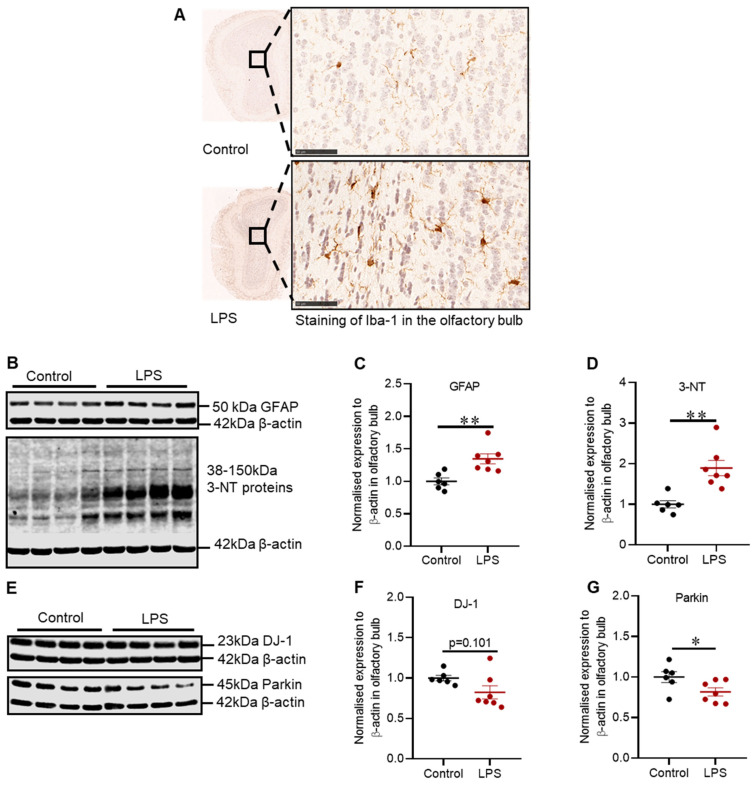
Intrastriatal administration of LPS altered the expression of inflammatory and oxidative stress markers and proteins involved in defence mechanisms against oxidative stress in the olfactory bulb of C57BL/6 mice. (**A**) Representative images for immunohistochemical detection of Iba-1 in the olfactory bulb. (**B**) Representative immunoblots for GFAP and 3-NT proteins in the olfactory bulb. (**C**) Densitometric analysis of GFAP protein. (**D**) Densitometric analysis of 3-NT protein. (**E**) Representative immunoblots for DJ-1 and parkin in the olfactory bulb (involved in defence against oxidative stress). (**F**) Densitometric analysis of DJ-1 protein. (**G**) Densitometric analysis of parkin protein. The results are presented as mean ± SEM (control n = 6; LPS n = 7). Statistical analysis was performed with a Mann–Whitney test, *p* < 0.05 (*), *p* < 0.01(**). Immunohistochemical detection of Iba-1 (scale bar: 50 µm; microscopic magnification: X400) (control n = 5; LPS n = 5).

Intrastriatal LPS has induced inflammation in the olfactory bulb illustrated by increased expression of Iba-1 positive microglia cells in the granular cell layer (Figure 5A), and this finding coincided with the increased expression of GFAP protein (1.34-fold) in the olfactory bulb (*p* = 0.005) (Figure 5C).

Inflammation in the olfactory bulb was accompanied by no significant reduction in DJ-1 protein (*p* = 0.101) (Figure 5F), but a 1.22-fold decrease in the expression of Parkin protein (*p* = 0.035) (Figure 5G) and a 1.89-fold increase in 3-NT proteins (*p* = 0.002) (Figure 5D).

### 2.6. Intrastriatal Administration of LPS Altered the Expression of Brain-Derived Neurotrophic Factor Protein in the Olfactory Bulb but Not in the Striatum of C57BL/6 Mice

**Figure 6:**
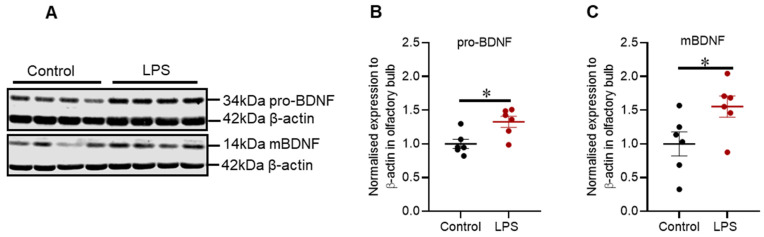
Intrastriatal administration of LPS altered the expression of pro-BDNF and mBDNF in the olfactory bulb of C57BL/6 mice. (**A**) Representative immunoblots for pro-BDNF and mBDNF in the olfactory bulb. (**B**) Densitometric analysis of pro-BDNF protein. (**C**) Densitometric analysis of mBDNF protein. The results are presented as mean ± SEM (control n = 6; LPS n = 6–7). Statistical analysis was performed with a Mann–Whitney test, *p*< 0.05 (*).

Brain-derived neurotrophic factor (BDNF) is synthesized in its precursor form (pro-BDNF), and it is enzymatically cleaved to generate the mature form (mBDNF). Intrastriatal administration of LPS increased protein expression of pro-BDNF by 1.27-fold (*p* = 0.015) (Figure 6B) and mBDNF by 1.44-fold (*p* = 0.041) (Figure 6C).

**Figure A2:**
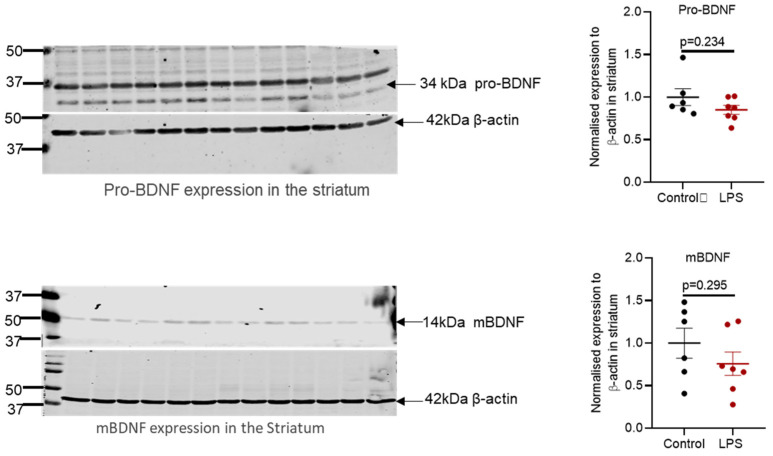
Western blot detection of precursor brain-derived neurotrophic factor (pro-BDNF) and mature brain-derived neurotrophic factor (mBDNF) in the striatum.

Additionally, the protein expression of pro-BDNF and mBDNF was assessed in the striatum via Western blot, but the proteins mentioned were not altered (refer to Figure A2 in Appendix A).

### 2.7. Intrastriatal Administration of LPS Induced Mild Inflammatory Changes and Alteration in Oxidative Stress Markers in the Distal Colon of C57BL/6 Mice

**Figure 7:**
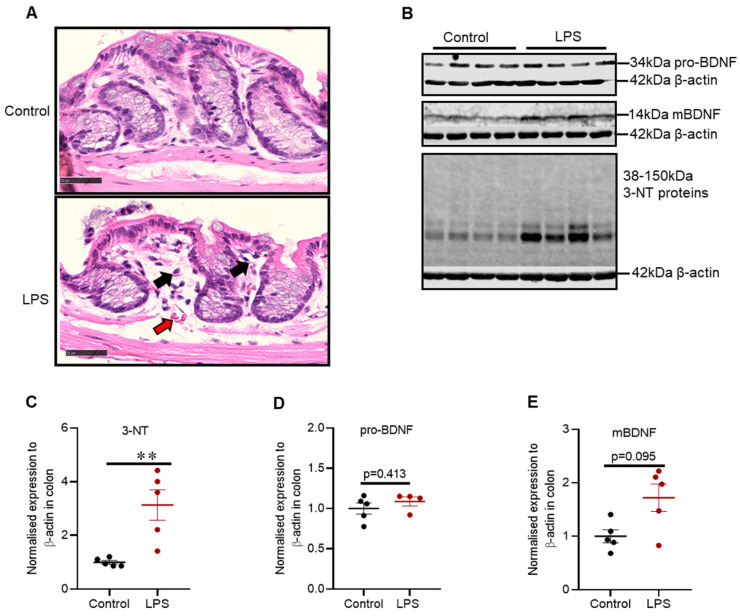
Intrastriatal administration of LPS induced mild inflammatory changes and alteration in oxidative stress markers in the distal colon of C57BL/6 mice. (**A**) Light microscopy of mouse distal colon stained with Hematoxylin and Eosin, whereby control mice showed intact epithelium lining with distinct muscularis mucosa, and LPS treated mice showed intact epithelium lining with a slight increase in mucosal vasculature (red arrow) and lymphocytes (black arrow) (Scale bar = 50μm, microscopic magnification: X400). (**B**) Representative immunoblots for 3-NT, pro-BDNF and mBDNF proteins. (**C**) Densitometric analysis of 3-NT proteins. (**D**) Densitometric analysis of pro-BDNF. (**E**) Densitometric analysis of mBDNF. The results are presented as mean ± SEM (control n = 5; LPS n = 4–5). Statistical analysis was performed with a Mann–Whitney test, *p* < 0.01(**).

Hematoxylin and Eosin staining for distal colon indicated that control mice had intact epithelium lining with distinct muscularis mucosa. In contrast, LPS treated mice showed intact epithelium lining with a slight increase in mucosal vasculature (red arrow) and lymphocytes (black arrow) (Figure 7A).

There was a 3-fold increase in the expression of 3-NT proteins (*p* = 0.008) (Figure 7C); however, intrastriatal administration of LPS did not alter the expression of pro-BDNF (*p* = 0.413) (Figure 7D) or mBDNF in the distal colon (*p* = 0.095) (Figure 7E).

## 3. Discussion

### 3.1. Main Findings of the Study

Intrastriatal administration of LPS induced motor impairment in C57BL/6 mice at 8 weeks post-treatment. This phenotype could be a consequence of LPS induced inflammation in the striatum, driving the pathological changes observed in this region, including degeneration of dopaminergic neuronal fibres, reduction of synaptic proteins, and decreases in DJ-1 and Parkin, the proteins involved in defence against oxidative stress. Moreover, intrastriatal LPS induced inflammation within the olfactory bulb, leading to alterations in synaptic proteins, but this did not induce a significant impairment in olfactory function. Thirdly, intrastriatal LPS induced mild inflammatory changes in the distal colon, which was accompanied by increased 3-NT proteins (refer to Table 1).

### 3.2. Motor Function and Striatal Pathology

LPS induced inflammation in the striatum, which was characterized by an increased number of Iba-1 positive microglia cells and GFAP positive astrocytes/GFAP protein, consistent with other neurotoxin models of PD [29,32,37,38]. This event was accompanied by a significant decrease in DJ-1 protein, but no significant alteration in Parkin. There were also no changes in 3-NT formation (a marker of oxidative stress). Prolonged inflammation mediated by microglia cells and astrocytes results in excessive production of inflammatory cytokines, chemokines and free radicals, which are detrimental to dopaminergic neurons and such pathological process is combated by the antioxidant system. Aside from their role in synaptic function, DJ-1 and Parkin have a key role in protection against oxidative stress via quenching of reactive oxygen species and clearance of damaged mitochondria, respectively. Thus, LPS- induced inflammation could have reduced the expression of DJ-1 protein, and in return, reduced DJ-1 could have enhanced susceptibility to LPS induced oxidative stress. Additionally, LPS did not alter the expression of 3-NT proteins. 3-NT is a marker of nitric oxide-dependent post-translation modification. Thus, it is possible that other indicators of oxidative stress that were not examined in this study could be changed such as the level of specific reactive oxygen species and antioxidants. Furthermore, intrastriatal LPS induced degeneration of dopaminergic terminals in the striatum illustrated by reduced immunoreactivity of TH positive cell terminals and TH protein; thus, providing consistent results with the previous in vivo studies [32,37,39]. Dopaminergic neurons are susceptible to cytotoxic molecules release via pathological activation of microglia cells and astrocytes; thus, degeneration of dopaminergic terminals may be a consequence of LPS induced inflammation [17,18,29]. Degeneration of dopaminergic synaptic terminals interferes with synaptic processes in the striatum, and coordination of motor function at the cortical level, which is consistent with impaired motor function observed in LPS mice and other in vivo studies [31,32]. The rotarod test indicated that LPS induced motor impairment at 8 weeks post-treatment, which was consistent with the TH loss in the striatum. Intrastriatal LPS did not induce a significant change in TH protein in the SN, a marker of dopaminergic neuronal integrity, inconsistent with Hunter and colleagues [32]. However, Hunter et al. in 2009 administered 20 µg of LPS bilaterally compared to our treatment regimen (10 µg unilaterally), and this could dictate the rate of progression of intrastriatal pathology to the SN, and resultant degeneration. Thus, our model is milder, and as a result, did not cause the SN degeneration. A longer time than 8 weeks may be required for the neurodegeneration to progress to the SN in our model. Administration of LPS reduced the levels of VMAT2 and SNAP-25 accompanied by increased α-synuclein protein in the striatum in our study. Defects in VMAT2 have been reported in patients and animal models of PD; however, the expression of SNAP-25 and VAMP2 proteins has not been widely examined in animal models of PD [12,13,40]. In our study, the reduction of VMAT2 and SNAP-25 proteins in the striatum could be due to the loss of dopaminergic terminals in this region as shown by the loss of TH. In addition, α-synuclein protein directly interacts with VAMP2 to promote the assembly of the SNARE complex; however, an increase in α-synuclein protein is evident to disrupt the release of neurotransmitters via defective clustering of synaptic vesicles [8,9]. Thus, an increase in α-synuclein protein and reduction of VMAT2 and SNAP-25 in our study could reduce dopamine release into the striatum and compromise neuronal transmission to the motor cortex, which can affect the control of movement. Furthermore, in addition to the increased α-synuclein protein expression, phosphorylated and aggregated forms have been identified in human PD [41,42]; therefore, future studies are required to investigate if similar changes occur in this model. Collectively, LPS induced inflammation in the striatum has led to the degeneration of dopaminergic fibres, reduction in synaptic proteins and DJ-1 protein, and these pathological processes could be responsible for motor impairment observed in C57BL/6 mice.

### 3.3. Olfactory Function and Pathology

Hyposmia is evident in up to 90% of PD patients, and it is reported to precede the motor symptoms of PD [43]. Thus, understanding olfactory function and pathology could be invaluable for the diagnosis of clinical PD at the early stages. LPS induced inflammation in the striatum led to an inflammatory response in the olfactory bulb characterized by increased Iba-1 positive microglia cells in the granular cell layer and GFAP protein. Although neuronal connections are yet to be thoroughly established between the nigrostriatal pathway and olfactory bulb, the prominent increase of Iba-1 positive microglia cells in the granular layer associated with intrastriatal LPS administration implies that inflammatory cells could have migrated from the striatum via the rostral migratory stream to the olfactory bulb, and this pathway needs to be investigated. Additionally, activation of inflammatory cells leads to the secretion of free radicals, consistent with increased expression of 3-NT proteins in the olfactory bulb of LPS treated mice. 3-NT is a marker of peroxynitrite induced nitration of tyrosine residues, and peroxynitrite is a compound of nitric oxide and superoxide, which are common free radicals associated with inflammation. Interestingly, LPS induced a significant decrease in Parkin protein with no significant reduction in DJ-1 protein in the olfactory bulb, which is the opposite to the striatum where we found reductions in DJ-1, but not Parkin. Parkin protects against oxidative stress; thus, reduced Parkin protein coincided with increased 3-NT proteins; however, further studies are critical to elucidate the function of Parkin and DJ-1 proteins in the olfactory bulb in PD models.

The olfactory bulb is the first relay station of olfactory sensory information, and it has a large group of dopaminergic interneurons in its glomerular layer. The literature is unsettled regarding the function of dopaminergic interneurons in the olfactory bulb. Some studies proposed that dopaminergic neurons have an inhibitory role in olfactory neurotransmission and have reported an increase in the number of dopaminergic neurons in vivo and PD patients [44,45,46]. In contrast, other studies have reported that dopamine neurotransmission in the olfactory bulb promotes neurogenesis, a phenomenon unique to this region and critical in olfaction [47]. Neural stem and progenitor cells from the subventricular zone of the lateral ventricle are evident to migrate via rostral migratory stream to the olfactory bulb, whereby these cells differentiate and integrate into GABAergic and dopaminergic interneurons [47]. Intrastriatal LPS did not induce a noticeable change in the number of TH positive dopaminergic cells in the glomerular layer; however, there was a small but significant increase in TH protein expression when assessed by Western blots. Our findings suggest that LPS did not affect the number of TH positive cells, but increased the expression of TH protein in these cells, suggesting increased dopamine signalling. Concomitant with these findings, LPS increased the expression of pro-BDNF and mBDNF proteins in the olfactory bulb, which indicates the increased expression of the BDNF gene. 

The olfactory bulb is a junction whereby sensory olfactory neurons synapse with mitral/tufted cells that extend to secondary olfactory structures and intrastriatal LPS could disrupt synaptic transmission. Our results showed that intrastriatal administration of LPS significantly decreased VMAT2 with no significant reduction in SNAP-25 and VAMP2 proteins. Decreased VMAT2 protein could be associated with LPS induced inflammation and oxidative stress in the olfactory bulb as a pathological pathway to increase cytosolic dopamine, dopamine-induced oxidative stress, and reduced neurotransmitter release [48,49]. It has been reported that reduced VMAT2 protein can augment hyposmia [49]; however, synaptic communication in the olfactory bulb has not been widely examined in models of PD and requires further clarification. Cumulatively, intrastriatal LPS has induced inflammation within the olfactory bulb, leading to alterations in synaptic proteins, but this did not induce a significant impairment in olfactory function as indicated by the buried food-seeking test. These findings indicate that pathological alterations found in the olfactory bulb were not at the threshold to produce a significant change in olfaction.

### 3.4. Colonic Pathology

PD is associated with a constellation of gastrointestinal complications such as drooling, dysphagia, delayed gastric emptying and constipation, and some of these complications precede motor symptoms in PD [2,50]. The most common gastrointestinal complication of PD is constipation, and it can be associated with colonic and anorectal dysmotility [51]. Therefore, we aimed to explore the effects of the nigrostriatal lesion (via intrastriatal LPS) on the distal colon. Hematoxylin and eosin staining of the distal colon indicated that control mice had an intact epithelium lining with distinct muscularis mucosa while LPS treated mice showed intact epithelium with a mild increase in mucosal vasculature and lymphocytes, indicative of mild inflammation; these findings were congruent with the increase in 3-NT proteins. Collectively, these findings indicate that intrastriatal LPS induced mild colonic inflammation and oxidative stress, which can induce pathological changes in the colon; thus, congruent with in vivo studies utilizing intranigral lesion to the nigrostriatal pathway [52]. The mechanisms linking CNS pathology and gastrointestinal complications are poorly understood in PD. In vivo studies have shown that there is a bidirectional relationship between the nigrostriatal dopaminergic system and gut system via the vagus nerve; however, there is limited experimental evidence [52]. Interestingly, vagotomy alleviates the pathology of the nigrostriatal dopaminergic system induced via gut dysfunction, and gut pathology induced via nigrostriatal lesions; however, additional studies are critical to thoroughly understand the involved mechanisms [52,53]. 

Intrastriatal LPS did not alter the expression of mBDNF and pro-BDNF proteins in the colon. Colonic mBDNF is expressed in epithelial cells and neurons of the myenteric plexus [54], and it is proposed to be involved in the regulation of colonic motility and visceral hyperalgesia [54]. Moreover, it has been shown that colonic mBDNF protein is increased in patients with irritable bowel syndrome, and it correlates with disease severity. Thus, future models of PD with potent gut abnormalities could examine the involvement of colonic mBDNF in the inflammatory processes [54].

In conclusion, intrastriatal administration of LPS has induced inflammation not only in the striatum, but also in the olfactory bulb and within the distal colon. LPS induced inflammation was responsible for pathological changes observed in the striatum such as degeneration of dopaminergic neuronal fibres, reduction of synaptic proteins, and proteins involved in defence against oxidants, and these pathological changes produced a motor phenotype similar to PD. Moreover, LPS has induced inflammation within the olfactory bulb, which caused alteration in synaptic proteins, but did not induce significant impairment in olfactory function. Our model recapitulated various aspects of human PD; thus, it could be useful for understanding the role of inflammation in motor and non-motor symptoms and associated pathology in PD. Future studies could modify our treatment regimen by utilizing bilateral intrastriatal injection of LPS (10 µg per hemisphere) to establish a greater lesion to the nigrostriatal pathway, which could better resemble the symptomatic stage of PD. Intrastriatal administration of LPS has induced changes in the olfactory bulb, striatum, and colon; however, we did not thoroughly explore the mechanisms of how LPS induced degeneration in each of the regions mentioned and neuronal connections between the regions, and this is a limitation. Moreover, reduced sense of smell and gastrointestinal complications (e.g., constipation) are common early non-motor symptoms in PD patients. Our study suggests that localized lesion to the nigrostriatal pathway induces pathological changes in the olfactory bulb and colon, which could be associated with the onset of olfactory and gastrointestinal complications. Therefore, future studies could extensively investigate early non-motor symptoms of PD such as impaired olfaction, impaired sleep-wake cycle, and gastrointestinal complications in the intrastriatal LPS model to establish their relationship with nigrostriatal lesions. Future studies could utilize tests such as habituation/dishabituation test for olfactory deficits, solid gastric emptying, and one-hour stool collection as measures of gastroparesis and colon motility, respectively, and finally, sleep latency to behavioural signs of sleep followed by polysomnography/electromyography to characterize alteration in the sleep-wake cycle [49,55]. This approach could help to establish a relationship between early non-motor symptoms of PD and lesions to nigrostriatal pathway and onset of motor symptoms; thus, it could aid in early diagnosis of PD and implementation of timely treatment.

## 4. Materials and Methods

### 4.1. Animals

This research was approved by the Animal Ethics Committee of the University of South Australia. Twenty-seven, 12 weeks old, C57BL/6 male mice were purchased from Animal Resources Centre, Western Australia and were housed at Core Animal Facility at the University of South Australia for the duration of the experiment. The mice were housed in groups of 3–4 in a pathogen-free environment at a room temperature of 22 °C, with a 12 h alternating light/dark cycle, and had access to food and water ad libitum.

### 4.2. Experimental Design

Mice were randomly allocated into two groups, a control group to be given phosphate-buffered saline (PBS) (n = 13) and an LPS group (n = 14). Each mouse was deeply anaesthetized through inhalation of isoflurane (3–4% isoflurane for induction, and 1–2% for maintenance) and mounted onto the stereotaxic frame (Stoelting, Wood Dale, IL, USA). The skin on the cranium was cleaned with 2% chlorhexidine/70% ethanol, and an incision was made on the scalp with surgical scissors to expose the cranium. 3% hydrogen peroxide was applied to the exposed cranium to easily define the bregma point. The following coordinates were used, starting from the bregma point to locate two injection sites in the right striatum: point A: +1.2 mm anterior-posterior, –1.5 mm medial-lateral, 3.5 mm deep, and point B: –0.34 mm anterior-posterior, +2.5 mm medial-lateral, and 3.2 mm deep. A fine needle was then used to drill a hole at each of the striatal injection sides. A 30 gauge 10 µL Hamilton syringe containing 1 µL of PBS for control mice or LPS solution (5 μg/µL LPS, Sigma-Aldrich, St. Louis, MI, USA) was slowly lowered ventrally to each of the injection sites and left in place for 2 min. The solution was slowly injected, and the needle was left in place for additional 2 min before it was gently withdrawn. After completion of the injection, 100 μL of a local analgesic mixture of lignocaine (2.5 mg/mL) and bupivacaine (0.63 mg/mL) was applied to the surgical wound, and the two ends of the scalp were glued together with surgical glue (Lyppard Australia Pty Ltd, Adelaide, South Australia, Australia). Each mouse was given 500 μL of sterile saline (0.9% sodium chloride) subcutaneously and kept warm on the heat pad to aid recovery post-surgery. 

**Figure 8:**
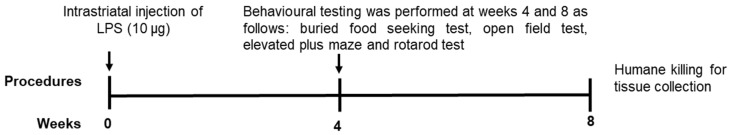
Timeline for intrastriatal administration of LPS in C57BL/6 mice. LPS was administered on day 1, followed by behavioural testing at 4 and 8 weeks post-treatment. The tissues were collected for subsequent analyses after the last behavioural test.

Behavioural testing was then conducted post-surgery at 4 and 8 weeks as outlined in Figure 8. Subsequently, the mice were humanely killed, and tissues were collected for biochemical and immunohistochemical analyses after the last behavioural test. 

### 4.3. Behavioural Testing

The mice were housed on a 12 h light/dark cycle, lights off at 7:00 p.m., and all behavioural tests started at 9:00 a.m. on the day of the test. Behavioural testing was conducted at 4 and 8 weeks after the injection of LPS as depicted in Figure 8. 

#### 4.3.1. Buried Food-Seeking Test

The buried food-seeking test is used to measure olfactory function in mice, and it is based on the ability of mice to use olfactory cues for foraging [56,57]. Before the test, the mice were fasted overnight for 14 h and then were allowed to individually search for a standard food pellet buried beneath 4 cm of bedding in an individually cleaned home cage. The mice were given a maximum time of 10 min to complete the task. An increase in time taken to find the buried food, which is referred to as latency time, is associated with olfactory impairment [57].

#### 4.3.2. Open Field Test

Open field test assesses both voluntary movement and anxiety in mice [58]. After acclimatization for 10 min, each mouse was placed in the centre of an open field arena (40 cm length × 40 cm width × 40 cm height), and its activity was tracked over 5 min using an overhead camera connected to ANY-maze, a video tracking software (ANY-maze version 7.01, Stoelting, Wood Dale, IL, USA). Mice have a natural aversion to open spaces, but are also explorative in their environment; therefore, a decrease in time spent in the central zone is a characteristic of anxiety-like behaviour [59].

#### 4.3.3. Elevated Plus Maze

Elevated plus maze test is used to assess anxiety-like behaviour, and it is based on rodents’ aversion to open spaces [60]. This repugnance for open spaces results in thigmotaxis, which refers to the avoidance of open spaces by confining movements to enclosed arms or the edges of bounded spaces. The test setup consists of a plus-shaped apparatus with two open and enclosed arms elevated at 40–70 cm from the floor. Each of the mice was placed in the central open area of the apparatus and were allowed to explore for 5 min. The movement of the mice was tracked using ANY-maze software. A significant decrease in time spent in the open arm of the maze is indicative of anxiety-like behaviour.

#### 4.3.4. Rotarod Test

Motor impairment is the major characteristic of clinical PD, and this feature was assessed in mice using the rotarod test, which utilizes a rotating rod to assess balance and coordination [61,62]. The mice were given 10 min before the test to acclimatize to the test room. The mice were then placed in the correct orientation on the rotating rod (3 cm diameter), and the rotarod apparatus (Ugo Basile, Gemonio, Varese, Italy) was set to accelerate from 5–30 rpm for 5 min. The test was ended when the mice fell off the rotating rod, swung 360° on the rotating rod instead of walking, or reached the maximum time of the test, and this time was recorded as latency time. The latency time of a given mouse was an average of three trials. The mice were given at least 10 min of rest between each trial. A decreased latency time is associated with motor impairment [61,62].

### 4.4. Fresh Tissue Collection and Homogenisation

Mice were sacrificed 24 h after completion of the rotarod test via an intraperitoneal injection of 60 mg/kg of sodium pentobarbitone, and the following fresh tissues were collected for biochemical analyses: olfactory bulb, striatum, substantia nigra/ventral tegmental area and distal colon. The tissues were homogenized in RIPA buffer (50 mM tris, 150mM sodium chloride, 1mM Ethylenediaminetetraacetic acid, 0.5% Triton X-100, 0.5% Sodium deoxycholate, pH 7.4) plus cocktail protease inhibitor (Sigma-Aldrich) using Precellys 24 Homogeniser (Bertin Technologies, Montigny-le-Bretonneux, France). Homogenates were centrifugated at 13,000 rpm for 30 min, and the supernatants were collected. The protein concentration of the supernatants was measured with a Micro-BCA^TM^ protein Assay kit (Thermo-scientific, Rockford, IL, USA) according to the manufacturer’s guidelines.

### 4.5. Western Blot

Proteins were separated by gel electrophoresis on 10–14% SDS-polyacrylamide gels using the CBS gel system (C.B.S Scientific, San Diego, CA, USA) for 90 min at 110 volts. The proteins were then transferred onto a 0.2 or 0.45μm nitrocellulose membrane (GE Healthcare Australia Pty Ltd, Sydney, New South Wales, Australia) at 0.6 amps for 90 min. The blots were air-dried for 90 min to enhance attachment of proteins to the nitrocellulose membrane before blocking with 5% bovine serum albumin (BSA)/tris buffered saline-tween (TBST) +0.05% azide or 5% skim milk/TBST + 0.05% azide (Sigma-Aldrich). After blocking, the membranes were incubated overnight at 4ºC with respective antibodies (refer to Table A1 in Appendix A). Following primary antibody incubation, the blots were washed with TBST and then incubated with corresponding secondary antibodies for near-infrared Western blot detection (Li-Cor Biosciences, Lincoln, NE, USA) for 1 h at room temperature. Immunoblots were visualized using Odyssey CLx imaging system (LI-COR Biosciences) and quantified with Image Studio Lite 5.2 (LI-COR Biosciences). Protein normalization was performed with mouse anti-β-actin [63].

### 4.6. Immunohistochemistry

Each mouse (control n = 5; LPS n = 5) was deeply anaesthetized via an intraperitoneal injection of 60 mg/kg of sodium pentobarbitone, and an incision was made into the abdominal cavity to expose the heart. Transcranial perfusion with 10% formalin was performed before brain collection. Subsequently, collected brains were fixed in 10% formalin for 48 h followed by tissue processing, paraffin embedding and sectioning of the paraffin blocks (4 µm). For staining, sections were deparaffinized in 2 changes of xylene and 2 changes of 100% ethanol for 5 min each before blocking endogenous peroxidase activity with 0.5% hydrogen peroxidase in methanol for 30 min. Antigen retrieval was carried out in a microwave using 10 mM sodium citrate buffer, then sections were blocked for non-specific binding using 3% normal horse serum (NHS) followed by overnight incubation with specific primary antibodies ((TH (Sigma-Aldrich) 1:3000 in 1% NHS + PBS/0.3 triton-X-100, GFAP (Dako, Denmark) 1:1000 in 1% NHS + PBS/0.3 triton-X-100 and Iba-1 (Wako, USA) 1:2000 in 1% NHS + PBS/0.3 triton-X-100)). Subsequently, sections were incubated with corresponding secondary antibodies (biotinylated anti-rabbit and mouse, 1:250 in 1% NHS + PBS/0.3 triton-X-100, Vector Laboratories Inc, CA, USA) for 30 min, incubated with streptavidin horseradish peroxidase-conjugated (Vector Laboratories Inc, CA, USA, 1:500 in 1% NHS + PBS/0.3 triton-X-100) for 1 h, and developed with 3,3′-diaminobenzidine (DAB) solution (0.05% DAB Sigma-Aldrich) and 0.015% hydrogen peroxide in 1X PBS) for 7 min. Sections were then counterstained with Mayer’s hematoxylin (Sigma, St. Louis, MI, USA) for 1 min, rinsed with water, differentiated in acid alcohol, rinsed with water, blued in 0.04% ammonia water, and lastly dehydrated, cleared and coverslipped using DPX mounting solution (Sigma-Aldrich). Images of the sections were taken using NanoZoomer S60 (Hamamatsu Photonics, Hamamatsu, City Shizuoka, Japan).

### 4.7. Hematoxylin and Eosin Staining for Distal Colon

Each mouse (control n = 5; LPS n = 5) was deeply anaesthetized via an intraperitoneal injection of sodium pentobarbitone (60 mg/kg), and an incision was made into the abdominal cavity to expose the intestines. Subsequently, the colon was dissected, flushed with cold PBS to remove intestinal content, and then divided into proximal and distal segments. The distal segment was further divided into two halves, one half was freshly frozen, and the other half was longitudinally cut open and assembled flat in a cassette for fixation (10% formalin). The tissues were fixed in 10% formalin for 24 h, processed using Leica ASP300 automated processor for 6 h and embedded in paraffin for sectioning. Subsequently, the tissues were sectioned at 4 µm with Thermo Scientific Microm HM 325 Rotary Microtome (Thermo Scientific). Hematoxylin and Eosin staining was conducted using Leica ST5010 Autostainer XL (Leica Biosystems, Melbourne, Victoria, Australia) and the histological slides were coverslipped using Leica CV5050 Fully automated Glass Coverslipper. Images of the sections were taken using NanoZoomer S60 (Hamamatsu Photonics).

### 4.8. Statistical Analysis

Behavioural and Western blot data were analysed with GraphPad Prism Software 8 (San Diego, CA, USA), and the results are presented as mean ± standard error of the mean (SEM). Statistical analyses for behavioural tests were performed with a two-way ANOVA test. Biochemical analyses comparing the control and LPS group were performed with a Mann–Whitney test. A statistical significance is reached when *p*-value is ≤0.05.

## Figures and Tables

**Table 1:** Summary of the proteins examined by Western blot. Downwards (↓) and upwards (↑) arrows indicate the direction of “significance”, left-right arrow (↔) refers to “no significance”, and dash (-) refers to “not assessed”.

	Changes in Protein Expression		
Proteins	Striatum	Olfactory Bulb	Colon
TH	↓	↑	-
α-syn	↑	↔	-
VMAT2	↓	↓	-
SNAP-25	↓	↔	-
VAMP2	↔	↔	-
GFAP	↑	↑↑	-
DJ-1	↓	↔	-
Parkin	↔	↓	-
3-NT proteins	↔	↑↑	↑↑
Pro-BDNF	↔	↑	↔
mBDNF	↔	↑	↔

## Data Availability

Not applicable.

## Appendix A

**Table A1:** Antibodies used for Western blotting in this study.

Primary Antibodies	Company	Dilution
Mouse anti-3-nitrotyrosine (3-NT)	Abcam	5% milk/TBST (1:3000)
Mouse anti-parkin	Santa Cruz Biotechnology	5% BSA/TBST (1:500)
Mouse anti-protein deglycase DJ-1	Santa Cruz Biotechnology	5% BSA/TBST (1:1000)
Mouse anti-synaptosomal associated protein 25 (SNAP-25)	Santa Cruz Biotechnology	5% BSA/TBST (1:1000)
Mouse anti-tyrosine hydroxylase (TH)	Sigma	5% BSA/TBST (1:7000)
Mouse anti-vesicular monoamine transporter 2 (VMAT2)	Santa Cruz Biotechnology	5% BSA/TBST (1:1000)
Rabbit anti-α-synuclein C-20	Santa Cruz Biotechnology	5% BSA/TBST (1:300)
Rabbit anti-glial fibrillary acidic protein (GFAP)	Dako	5% milk/TBST (1:2000)
Rabbit brain-derived neurotrophic factor (BDNF)	Osenses Australia	5% BSA/azide (1:1500)
Rabbit precursor brain derived neurotrophic factor (pro-BDNF)	Osenses Australia	5% BSA/azide (1:3000)
Rabbit anti-vesicle associated membrane protein 2 (VAMP2)	Osenses Australia	5% BSA/TBST (1:1000)
Mouse β-actin	Abcam	TBST (1:15 000)
Secondary antibodies		
Donkey anti-rabbit IgG	Li-Cor Biosciences	TBST (1:20,000)
Goat anti-mouse IgG	Li-Cor Biosciences	TBST (1:20,000)
